# Radiation Metabolomics: Current Status and Future Directions

**DOI:** 10.3389/fonc.2016.00020

**Published:** 2016-02-02

**Authors:** Smrithi S. Menon, Medha Uppal, Subeena Randhawa, Mehar S. Cheema, Nima Aghdam, Rachel L. Usala, Sanchita P. Ghosh, Amrita K. Cheema, Anatoly Dritschilo

**Affiliations:** ^1^Department of Oncology, Georgetown University Medical Center, Washington, DC, USA; ^2^Department of Radiation Medicine, Georgetown University Medical Center, Washington, DC, USA; ^3^Lombardi Comprehensive Cancer Center, Georgetown University Medical Center, Washington, DC, USA; ^4^School of Medicine, Georgetown University Medical Center, Washington, DC, USA; ^5^Armed Forces Radiobiology Research Institute, Bethesda, MD, USA

**Keywords:** ionizing radiation, metabolomics, biomarkers

## Abstract

Human exposure to ionizing radiation (IR) disrupts normal metabolic processes in cells and organs by inducing complex biological responses that interfere with gene and protein expression. Conventional dosimetry, monitoring of prodromal symptoms, and peripheral lymphocyte counts are of limited value as organ- and tissue-specific biomarkers for personnel exposed to radiation, particularly, weeks or months after exposure. Analysis of metabolites generated in known stress-responsive pathways by molecular profiling helps to predict the physiological status of an individual in response to environmental or genetic perturbations. Thus, a multi-metabolite profile obtained from a high-resolution mass spectrometry-based metabolomics platform offers potential for identification of robust biomarkers to predict radiation toxicity of organs and tissues resulting from exposures to therapeutic or non-therapeutic IR. Here, we review the status of radiation metabolomics and explore applications as a standalone technology, as well as its integration in systems biology, to facilitate a better understanding of the molecular basis of radiation response. Finally, we draw attention to the identification of specific pathways that can be targeted for the development of therapeutics to alleviate or mitigate harmful effects of radiation exposure.

## Introduction

**Figure 1:**
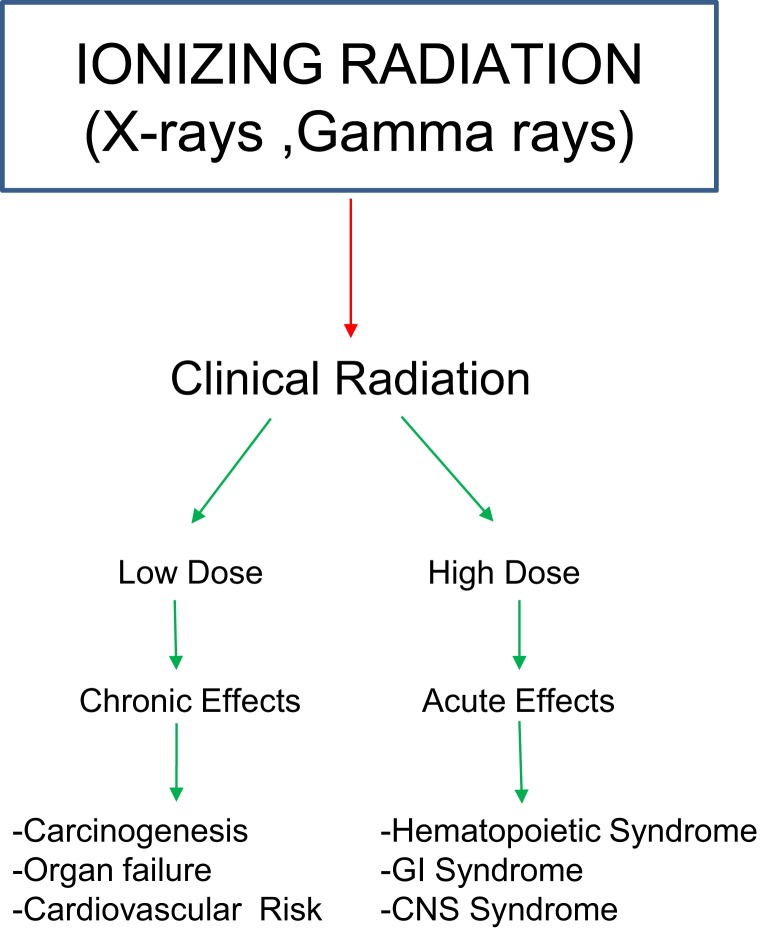
**Impact of exposure to ionizing radiation on biological systems**.

Exposure to ionizing radiation (IR) can cause deleterious effects in humans, dependent on dose and rate of exposure (Figure 1). Sub-lethal doses may cause few or no acute symptoms; however, longer term follow-up may reveal radiation-induced carcinogenesis, severely affecting quality of life of exposed personnel. Therefore, there is a need to develop biomarkers indicative of early and delayed whole body and organ-/tissue-specific injury that may facilitate the clinical management of afflicted populations.

Understanding therapeutic response to radiation is of critical clinical importance since approximately two-thirds of cancer patients receive radiotherapy (1). The Cancer Biology/Radiation Biology Task Force appointed by the American Society for Radiation Oncology (ASTRO) Board of Directors recently recommended the study of tumor metabolism, as well as tumor genomics and epigenetics, as promising areas for research for the advancement of radiotherapy treatment of cancer (2). Furthermore, the general population is subjected to low-levels of radiation due to environmental or occupational exposure on a routine basis (3). Inadvertent catastrophes such as the Fukushima disaster and the increasing risk of terrorism necessitate a diagnostic and monitoring platform that is easily deployable, reproducible, accurate, and rapid for evaluation of radiation exposure. Since metabolomics is a high-throughput technology, it is well suited for this goal and can be performed on readily accessible biological samples, such as urine or serum.

In the event of mass casualty incidents, biomarkers can offer a tool to triage used to segregate “at risk” population (4). Although nausea, vomiting, and erythema may manifest as acute radiation syndromes of 1–4 Gy, there is a latent period before the physiological signs develop (5). Employing metabolomics to analyze and quantify variations in concentrations of small molecule metabolites comprising the metabolome can help to identify the physiological status of an individual even before symptoms become apparent (6).

High thorough-put technologies and analyses have fueled novel scientific discoveries, but thus far, biological “big data” has failed to be translated to a real-world understanding of pathology phenotype profiling (7). The transition from “omic” bench-work to patient-bedside is complicated by biological processes that are subjected to regulatory mechanisms. Epigenetics, microRNA interference, and post-translational modifications of proteins are reflected in genomics, transcriptomics, and proteomics analysis and provide an indirect image of cellular phenotype while metabolomics can be used as a “read-out” of current physiology (7, 8). Since metabolites are not subject to many complex post-processing mechanisms, they are deemed to be closest to cellular phenotype and hence valuable for developing a robust band of biomarkers (8). Metabolomics has augmented discovery of biological biomarkers for pathway perturbations in cancer (8–11), neurological disorders (12, 13), cardiovascular disease (3, 4, 14–16), diabetes (5, 17, 18), and alcohol-induced liver injury (19). Finally, metabolomics has helped to elucidate the biology of treatment responses and environmental exposures (20–22).

Metabolomics is an emerging new discipline that identifies and quantifies small molecules (50–150 Da) that are downstream of genomic, transcriptomic, and proteomic processes. Use of this technology is fast gaining credence for the development of molecular signatures of various pathological condition and therapies. Metabolomics-based molecular profiling has been used successfully for assessing qualitative and quantitative response of exposure to IR. The field has also seen rapid and ongoing development of statistical tools for analyzing data from metabolomics profiling that is critical for drawing meaningful interpretation for clinical and translational applications.

Herein, we review current status of metabolomics technologies, data analytics, database utilization, and pathway analyses that are driving the advancement of this approach for developing biomarkers predictive of exposure to IR and concomitant risk of developing specific pathologies over time. We discuss the importance of metabolomics studies using cellular or tissue, rodent, and primate models in the context of radiobiology as well as the future of clinical and translational radiation research through a systems-wide integration and statistical modeling of metabolomics with recommendations for standardization of sample collection and data analysis processes for future studies.

## Metabolomics Technologies, Data Analysis, and Scope of Applications

The promise of metabolomics as a scientific tool has been fueled largely by the advancement in nuclear magnetic resonance (NMR) and mass spectrometry (MS). NMR is an analytical tool that utilizes the resonance absorption profiles of molecules in a magnetic field. MS generates profiles of mass to charge ratios from ionized molecules that are separated by a mass analyzer and detected by an ion detector (9, 10). The choice of technological platform for a particular experiment depends on the type of the available biological sample and its characteristics, the research question of interest, sensitivity and associated costs. Historically, NMR has been the platform of choice, because it is a standalone technology, samples required no processing (non-destructive technique), and it provides unambiguous structural information about metabolites (11). Additionally, NMR spectroscopy with magic angle spinning allows for the analysis of intact tissues (12). Although several techniques such as J-resolved, TOCSY, and HSQC spectra exist to enhance NMR sensitivity (13, 14), the dynamic range is not ideal for detection and identification of low abundance biomarkers. Over the last decade, MS has become increasingly popular due to its superior sensitivity compared to NMR mainly because of rapid advancements in resolution and sensitivity of the instruments that facilitate the detection of low abundance compounds. For instance, a typical analytical run using a C_18_ reverse chromatography in conjunction with time of flight MS yields around 5000–8000 features. Targeted MS approaches with optimization can achieve femtomolar sensitivities depending on the compound of interest. Although methodologies of direct infusion (DI) MS (without chromatographic separation) exist, more sensitive and high-throughput MS analysis is achieved by employing a chromatographic platforms including gas chromatography (GC), liquid chromatography (LC), capillary electrophoresis (CE), or supercritical fluid chromatography (SFC) in conjunction with high-resolution MS (9). Frequently, the use of a specific chromatographic method for resolving small molecules in complex biological mixture is based on the characteristics of the metabolites of interest. Typically, following chromatographic separation, small molecules are ionized using electrospray ionization and are resolved within the mass analyzer based on mass to charge ratio and get detected in real time. Development of a variety of ionization techniques (e.g., electron ionization, chemical ionization, fast atom bombardment ionization, electrospray ionization, and matrix-assisted ionization), mass analyzers (e.g., quadrupole, magnetic sector field, electric sector field, time of flight, and ion trap), and ion detectors (electron multiplier, multichannel plate, and Faraday cup) facilitate the analysis of small molecules metabolites that are known to be structurally and chemically diverse (15).

The analyses of NMR and MS metabolomics data share many common pre-processing and post-processing steps. Pre-processing describes conversion of raw spectral data into qualitative and quantitative information and involves such steps as outlier screening, baseline correction, transformation, normalization, and peak binning. The end-product of NMR pre-processing is a matrix of chemical shifts and intensities for samples, contrary to the end-product of MS pre-processing which is a matrix of mass to charge ratios, retention times, and abundance values for samples. However both NMR and MS post-processing of metabolomics data involve cleaning and analyzing the data, and translating the data to biologically relevant interpretations. Post-processing steps involve statistical analysis such as principal component analysis (PCA), support vector machines, and database query to make putative identifications (16–19).

## Effect of Radiation on Biomolecules

**Figure 2:**
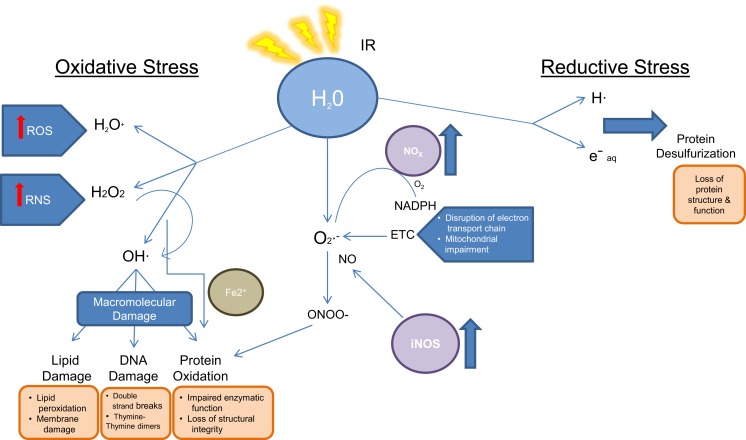
**Oxidoreductive and macromolecular damage as a consequence of ionizing radiation exposure**.

Radiation biology involves the study of effects of energy deposition by IR on biological systems and the subsequent cellular response and damage as a consequence of both direct and indirect effects of the radiation. Direct damage due to radiation is caused by breakage of specific bonds within 10^−14^ s of exposure in the biomolecules (S–H, O–H, N–H, and C–H). Indirect damage is related to water radiolysis and the rapid formation of reactive oxygen species (ROS), which occurs within 10^−12^ s of IR exposure. Subsequent formation of secondary ROS such as superoxide (${\text{O}}_{2}^{-}$), hydrogen peroxide (H_2_O_2_), and reactive nitrogen species (RNS) such as peroxynitrite anion (ONOO^−^) and peroxynitrous acid (ONOOH) also cause further damage to DNA and the cellular compartments, misbalance the stoichiometry of biochemical reactions, and perturb functionally relevant pathways, all within 1 ms of exposure (20–22). Furthermore, IR exposure induces the formation of endogenous ROS and RNS through mitochondrial electron transport chain, stress mechanisms, and elevates the expression of ROS producing enzymes. For instance, IR influences the overexpression of inducible nitric oxide synthase (iNOS), which leads to an increased level of NO, a precursor of ONOO^−^. Similarly, IR induces the expression of NADPH oxidase, which converts oxygen and NADPH to superoxide (${\text{O}}_{2}^{-}$) and hydrogen ion (23, 24). Increased NADPH oxidase levels can be observed months after irradiation. Together with renin–angiotensin system member peptide and its receptor (AT1R), NADPH oxidase produces ROS (25, 26). The generation of oxidants and reductants as a consequence of IR exposure subsequently results in biomolecule damage as illustrated in Figure 2 (27).

DNA is particularly vulnerable to ROS-induced damage, resulting in single-base damage, sugar damage, single- and double-strand breaks, DNA–DNA, and DNA–protein cross links. As a result, metabolites related to DNA damage and repairs have been frequently reported in biodosimetry studies using targeted and untargeted metabolomics approaches.

The ability of cells to survive after DNA damage lies in mobilizing oxidative stress-defense mechanisms. Initially, low-molecular weight endogenous antioxidants (e.g., thiols, glutathione, ascorbate, melatonin, lipoic acid Coenzyme Q10, Vitamin E, etc.) neutralize water radiolysis products, oxidized molecules, and peroxynitrite. Subsequently, levels of endogenous antioxidants fall rapidly, and enzymatic detoxification combats secondary elevated endogenous ROS as a secondary level of cellular protection. For instance, superoxide dismutases (SODs) convert ${\text{O}}_{2}^{-}$ to H_2_O_2_ and O_2_. H_2_O_2_ is then detoxified by oxyredoxins and glutathione peroxidases. Persistent oxidative stress causes activation of transcription factors, which bind to sequences that encode for detoxifying enzymes (e.g., SOD, GPx, glutathione *S*-transferase, heme oxygenase-1 among others) (28–32). These processes impact endogenous metabolites, for instance, increased oxidative stress leads to mitochondrial impairment causing a disruption in electron transport chain and oxidative phosphorylation. Additionally, lipid transport across the mitochondrial membrane is affected. Secondary causes of IR exposure result in membrane damage due to lipid oxidation and peroxidation events (33).

Nevertheless, depending on the level of damage, detoxification and protective processes of the cells are often compromised, leading to DNA damage. Activation of cell cycle checkpoints and DNA repair mechanisms follow multiple kinase cascades, DNA repair signaling, cell cycle arrest, and apoptosis. DNA damage response mechanisms are activated by sensor, transducer and effector proteins. The DNA damage sensor complex MRN (MRE11, Rad50, and NBS1) helps recruit ATM and ATR to the DNA damage sites. Downstream of their signal transduction are histone H2AX, checkpoint kinases Chk1 and Chk2, and adenosine monophosphate-activated kinase (AMPK). These signals activate important DNA damage response proteins and transcription factors such as p53, BRCA1, Nbs1, C-Abl, mTOR, p21Cip1, and p27kip1 (34, 35). Mediation of many of these components can result in cell cycle arrest at G1/S and G2/M and subsequent apoptosis. ATM, also known as the ataxia telangiectasia mutated gene, mediates phosphorylation of H2AX when there are double strand breaks in DNA, whereas the ATR gene mediates H2AX phosphorylation due to single strand breaks in S-phase arrested cells (36). Until recently, it was believed that H2AX activation is mostly mediated by ATM, but recent studies suggest that ATR is responsible for the majority of both early and late (after 24-h) responses to ROS even in non S-phase cells (36). However, mechanisms of this activation are still unclear. Interestingly, H2AX histone alterations have been found after low-dose and high-dose radiation exposure (34–37).

In addition to DNA, polyunsaturated fatty acids (PUFA) and other lipids that are integral components of cell membrane are highly susceptible to IR exposure damage (38, 39). Cellular damage involving lipids after IR also includes sphingolipids, especially ceramide (40). Ceramide is produced by sphingomyelinase enzyme, which relocates from lysosomes into the cytoplasm after IR. Excessive production of ceramide may lead to apoptosis as well as enlargement of lipid raft micro domains into lipid platforms; these enlargements are enriched in receptors, proteins, and nuclear factors, which changes intracellular signaling. Overall, IR-induced lipid alterations may cause increased membrane permeability, changes in ion gradients, additional radical generation, changes in signaling, and ultimately cell death. IR-induced protein damage is principally mediated by ROS, which can be monitored using MS techniques. For instance, hydroxyl ion initiates generalized breakage of protein backbone, although amino acids with aromatic rings are particularly vulnerable. On the other hand, IR-induced protein carbonylation is mostly specific to amino acids such as lysine, cysteine, histidine, threonine, proline, glutamate, asparagine, and arginine (41, 42). Perturbation of proteins by radiation exposure may cause signal transduction alterations, RNS formation, and damage of other biomolecules. Recently, techniques to improve radiation therapy, such as proton beam therapy have emerged, both to reduce normal tissue toxicity and to facilitate a more targeted approach. Proton beam therapy has been shown effective in treating small brain tumors, head and neck tumors, chordomas, due to an improved conformal delivery, permitting dose escalation. By virtue of lower off-target radiation exposures, proton beam therapy may be superior to conventional radiation for benign lesions, by reducing the risk of secondary malignancies (43). However, there is little research being done to understand how protons affect biological processes at molecular level.

## Metabolomics Studies in Cellular or Tissue Models

In an effort to complement transcriptomic and proteomic studies on radiation exposure, Patterson and colleagues conducted a study of radiation markers *in vitro* using a metabolomics approach. The differential generation of hydrophilic metabolomes in TK6 and BJ cell lines were studied over a pre-determined interval after radiation exposure using ultra-performance liquid chromatography with electrospray ionization time-of-flight mass spectrometry (UPLC-ESI-TOFMS). Interestingly, this group used an innovative visualization tool that had been developed for analysis of gene expression data by clustering closely related metabolomes. Predictably, the depleted metabolomes were found to be related to oxidative stress response and DNA damage. AMP levels were found to be significantly depleted 1 h post-radiation representing an acute effect. On the other hand, metabolites like glutathione, NAD^+^, and spermine showed significant differences at 1 h followed by normal levels at 8 h; however, by 16 h following radiation exposure, the endogenous levels of these metabolites were the same as that observed at 1 h post-radiation (44). In addition, response to radiation differed in the two cell lines emphasizing differential radio sensitivity.

In order to understand ATM-mediated DNA repair mechanisms, we have reported a study where isogenic cell lines were irradiated, and the cellular response was studied overtime. We used a hypersensitive cellular strain of ataxia telangiectasia fibroblasts, AT5BIVA, as well as second genetically engineered cell line (ATCL8), with the exogenously introduced wild-type ATM gene. Metabolomic profiling of irradiated AT5BIVA revealed dysregulated glycerophospholipid metabolism and phospholipid degradation. In contrast, metabolomic profiling of the ATM proficient line (ATCL8) revealed changes in abundance of biomolecules participating in many pathways including purine metabolism, linoleic acid metabolism, pentose and glucuronate interconversions, and fructose and mannose metabolism after irradiation. This study helped correlate alterations in radiation-induced metabolic responses based on a single-gene perturbation. Furthermore, a preponderance of proteomic evidence strengthened the conclusions drawn from metabolomics profiling (45).

With the help of CE mass spectrometry (CE-MS), Lee and Britz-McKibben identified metabolomes associated with radiation-induced apoptosis in human leukocytes (46). Flow cytometry allowed differentiation between apoptotic, non-apoptotic, and necrotic cells. Furthermore, staining in flow cytometry was used to differentiate early and late apoptotic cell lines. The correlation of flow cytometry with CE-MS data revealed up-regulation of arginine, glutamine, creatine, and proline levels, in comparison with reduced glutathione levels in irradiated versus sham-irradiated leukocytes. As discussed in the Patterson study, the metabolites identified in this study belong to pathways of oxidative stress and energy metabolism. Despite the diversity of methods used by the two studies, these cellular processes identified were indeed comparable. Nevertheless, further research is necessary to resolve the generality of these findings and their attribution to cellular response to radiation or to determine if these metabolites are non-specific markers of cellular response to stress.

Another study analyzed the effects of IR on surviving immune T cells from previously irradiated animals and the observed changes in the cellular metabolic profiles. Li and colleagues concluded that IR impaired the metabolic reprograming of activated T cells This led to a decrease in effectiveness of vital metabolic mechanisms needed for activation including, “glucose uptake, glycolysis, and the energy metabolism.” This approach could be used to investigate how transformations of T cells can be used as potential targets for combined modality therapeutic methods such as radiotherapy and immune therapy (47).

In another study, human keratinocytes when irradiated to a low dose (<10 cGy) exhibited time- and dose-related disruptions in DNA/RNA damage repair and lipid and energy metabolomic pathways. The difference in the levels of the metabolites showed a delayed response to the low-dose IR as the shift in the metabolite levels are different from controls at 48 h and not at previous time points. This response imitates the radiobiology of tissues irradiated to high doses, suggesting that biomarkers may be present even at low-dose radiation exposures (48).

## Metabolomics Studies in Rodent Models

Murine and rat models have been extensively used for radiation metabolomics studies since they can be performed under controlled conditions (age, gender, genotype, and diet) so as to derive radiation specific inter-subject metabolite comparison. Also, specimens from rodents (e.g., serum, urine, intestine or lung) are readily available. Some of the earliest metabolomics studies of radiation exposure in rodent models used urine as an analyte. Tyburski and colleagues used a targeted metabolomics approach to investigate β-thymidine and *N*-hexanoylglycine urinary biomarkers after radiation exposure (49). In this study of metabolomic characterization of radiation response, animal handling was found to be extremely important for the accuracy of the results; early in the study, one component of the mouse chow was erroneously identified as a marker of radiation sensitivity. Nevertheless, subsequent experiments under more controlled dietary and caging protocols revealed more conclusive results (50). Dose-related increase in urinary thymidine, 2**′**-deoxyuridine, 2**′**-deoxyxanthosine, xanthine, and xanthosine were observed in the mice-irradiated with sub-lethal dose of gamma rays. The metabolites identified by Tyburski and colleagues differed from more classical markers of radiation exposure related to oxidative damage to DNA structure (e.g., 8-hydroxy-2**′**-deoxy guanosine thymine glycol and thymidine glycol), suggesting that gamma radiation may damage DNA differently than other forms of radiation.

Untargeted metabolomics profiling and subsequent PCA analysis of rat serum 24-h post-irradiation with 0, 0.75, 3, and 8 Gy doses revealed nine serum markers of radiation exposure. Inositol, serine, lysine, glycine, threonine, and glycerol were upregulated, whereas isocitrate, gluconic acid, and stearic acid were down-regulated (51).

Another study used UPLC-ESI-TOFMS coupled with PCA for the analysis of serum from mice exposed to 3 Gy of radiation (a non-lethal dose) at 2, 4, 6, and 8 months of age; which demonstrated elevation of DNA damage biomarkers (e.g., thymidine, xanthosine, 2′-deoxyuridine) and N^1^-acetylspermidine. The levels of some metabolites such as 2′-deoxyuridine and xanthine did not get affected when exposed at long intervals to continuous irradiation. Findings from this study emphasize the role of polyamine metabolism toward impacting the efficiency of DNA damage and repair that shows a progressive decline with age. Prediction of exposed mice based on xanthosine and 2′-deoxyuridine urine metabolomic profile was robust (82 and 98%, respectively) irrespective of age and exposure history, which suggests a non-invasive signature for sub-lethal radiation exposure (52).

The use of metabolomic technology is likely to yield a panel of biomarkers, and not just a single molecule, that can be potentially used to identify the biological effects of radiation (53). Nevertheless, some metabolites may be identified by targeted metabolomics approaches as individual biomarkers. In a murine model study by Jones and colleagues, such an approach was utilized (54). Citrulline and retinoic acid were investigated as biomarkers for dose-related analysis of radiation damage to the intestine and lung, respectively. Three strains of mice exposed to 15 Gy of radiation to the lung showed abnormal lung histology and accompanying decrease in retinoic acid levels 24 h and 180 days post-irradiation with metabolomic quantification of lung tissue by LC-MS/MS. The group of mice exposed to full-body irradiation doses of 8, 9, and 10 Gy had a 50–60% decrease in plasma citrulline concentration 4 days post-irradiation compared to controls, whereas mice exposed to 11–15 Gy doses demonstrated a 90% decrease in plasma citrulline. At day 6, mice exposed to lower doses of radiation recovered circulating citrulline levels, whereas mice with 11–12 Gy levels of exposure had <50% of that of the control levels, and mice exposed to 13–15 Gy had no recovery in circulating citrulline levels. Intestinal histology did correlate with citrulline levels as well.

In another murine model study, radiation damage to intestinal tissues was investigated (55). Metabolomic profiles of mice at 1 and 4 days post sub-lethal irradiations at 4 and 8 Gy to identify markers of GI injury. Utilizing MS and multivariate analysis, lipids, glutamate, tryptophan, taurocholate, and the dipeptide Cys–Gly were identified as biomarkers of intestinal injury following irradiation. Using sophisticated statistical analysis, subsequent pathway analysis suggested dysregulation in arachidonic acid metabolism, eicosanoid signaling, and oxidative phosphorylation. One of the significant challenges of defining biomarkers specific to radiation exposure is rationalization of metabolomics findings in a biological context. This study exhibited the potential of using metabolomics for the identification of biomarkers indicative of tissue-specific injury.

Importantly, rodent models have also been used to explore the possibility that radiations of differing quality [high linear energy transfer (LET) compared to low LET] result in different metabolomics profiles. Metabolomic profiles of intestinal specimens from mice exposed to 137 cGy gamma irradiation versus an equitoxic ^56^Fe heavy ion irradiation exposure were evaluated with UPLC-QTOF-MS techniques (56). Biological relevance of both radiation exposures was evaluated using pathway analysis, immunoblots, and immunohistochemistry. Although exposures to both types of radiation resulted in perturbed amino acid metabolism profiles, PCA and OPLS-DA analysis suggested that metabolomic profiles differed. Specifically, ^56^Fe radiation preferentially affected dipeptide metabolism and resulted in elevation of prostanoid biosynthesis and eicosanoid signaling that is involved in cellular inflammation implicated in bowel disease.

It is anticipated that radiation metabolomics may eventually be used to study radiation damage as well as to identify biomarkers related to successful radiotherapy response. In a mouse xenograft model of human pancreatic cancer, high-resolution magic angle spinning proton magnetic resonance spectroscopy was used to monitor metabolomic changes in tumor specimens before and after radiotherapy. Tumor growth was inhibited proportionally to three increasing doses of radiation. The exposure to escalating radiation dose resulted in an increase in choline, taurine, alanine, isoleucine, leucine, valine, lactate, and glutamic acid in the tumor tissue whereas a depletion of endogenous phosphocholine, glycerophosphocholine, and betaine were observed (57). These results indicate that there is potential for the use of radiation metabolomics in the therapeutic fields for early detection and treatment of cancer.

## Clinical Studies Using the Metabolomics Approach

Blood or urinary metabolomics is a promising biomarker discovery platform owing to their minimally invasive properties. Radiation metabolomics using clinical samples are constrained by ethical considerations, such that majority of investigations have focused on the use of a controlled, generalized radiation exposure that can only be performed in the laboratory setting on animals rather than on human subjects. Primates are considered the closest animal model to humans. Thus, radiation metabolomics studies on non-human primates are a valuable surrogate. Using UPLC-ESI-QTOFS MS, Johnson and colleagues used untargeted and targeted metabolomics techniques to analyze urine from rhesus monkeys exposed to various gamma radiation (1, 3.5, 6.5, 8.5 Gy) at various time points within 3 days post-irradiation (58). As many as nine novel and robust markers of IR in non-human primates (such as adipic acid) were identified. Some biomarkers identified in the rhesus model were shared by mouse and rat irradiation biomarker models (such as *N*-acetyltaurine and isethionic acid). Identified biomarkers of irradiation in the non-human primate model were metabolites belonging to DNA damage, taurine metabolism, and creatine and creatinine pathways. Inter-species comparison of biomarkers of radiation exposure picked out taurine as a common metabolite between the mouse, rat, non-human primate, and humans and demonstrated similar metabolite profile between the same species, such as the humans and non-human primate (metabolites such as carnitine, acetyl carnitine, hypoxanthine, creatine) and the mouse and the rat model (metabolites such as thymidine, *N*-hexanoylglycine, 2′-deoxyxanthosine, 2′-Deoxyuridine) (Table 1) (49, 50, 58–62). Even though other studies have reported several radiation biomarkers, the limitation of converting biomarker panels from discovery to clinical application requires a more concerted effort. One of the biggest challenges is the lack of preclinical biomarkers that can detect risk of IR exposure before the appearance of clinical symptoms (63). One way to overcome this is to combine several biomarkers from different “omics” assays to improve the sensitivity of the assays or techniques we use for the detection. Additionally, we can improve the study design by stratifying sub groups (63).

**Table 1:** **Inter-species comparison of metabolites of radiation exposure that can vary with respect to dose, type, and time**.

Metabolite	Observed change	Species	Reference
3-Hydroxy-2-methylbenzoicacid	↑	Rodents	(48)
3-*O*-sulfate	↑		(48)
SAA levels	↑		(59)
Xanthosine	↑		(49)
*N*-Hexanoylglycine	↑		(60)
2′-Deoxyxanthosine	↑		(57)
2′-Deoxyuridine	↑		(57)
Thymidine	↑		(57)
*N*^1^-Acetylspermidine	↑		(60)
Glyoxylate	↑		(61)
Threonate	↑		(61)
p-Cresol	↑		(61)
*N*-Acetylglucosamine/galactosamine-6-sulfate	↑		(60)

Tyrosol sulfate	↑	Non-human primate	(57)
3-Hydroxytyrosol sulfate	↑		(57)
*N*-Acetylserotonin sulfate	↑		(57)
Tyramine sulfate	↑		(57)
Adipic acid	↑		(57)
Creatinine	↑		(57)
Creatine	↑		(57)

Tri methyl-I-lysine	↓	Human	(58)
Decanoylcarnitine	↑		(58)
Octanoylcarnitine	↓		(58)

Xanthine	↑	Rodents/non-human primate/human	(57)
Taurine	↑		(57)

Acetylcarnitine	↓	Non-human primate/rodents	(58)
Hypoxanthine	↑		(57)
Uric acid	↑		(57)

*N*-Acetyltaurine	↑	Non-human primate/rodents	(57)
Isethionic acid	↑		(57)

The first radiation metabolomics study of human urine post-exposure to full body radiation of patients undergoing radiotherapy was published recently (59). Ultra-performance LC combined with time-of-flight mass spectrometry (TOFMS) was used to analyze urine of patients who had received total body irradiation before undergoing hematopoietic stem-cell transplantation. Of the seven markers that showed differential expression pre- and post-­irradiation, several were identified as players in the transport of fatty acids across mitochondria for consequent fatty acid β-oxidation. Other metabolites identified as differentially expressed in irradiated samples have been associated with increased oxidative stress and radiation-induced DNA damage. Interestingly, gender differences were also present in the post-irradiated samples.

However, biomarkers of radiation exposure that have been derived from human subjects so far may be confounded by cancer-specific markers, which may not be generalizable to “healthy” subjects exposed to larger or longer duration doses of radiation. Nevertheless, radiation oncology may gain much from radiation metabolomics studies. Metabolomic studies of cancer patients may help determine appropriate doses for radiotherapy and for disease prognosis (64). Wibom and colleagues sampled the intracranial fluid of patients with glioblastoma multiforme (GBM) after radiation therapy in areas of tumor and healthy tissue surrounding the tumor using a micro-dialysis catheter (65). GC-MS metabolomics profiling revealed many differences between healthy and tumor tissue profiles. The extensive panel of markers had ROC values of 0.896 and 0.821 for tumor and healthy brain tissue, respectively. The invasive nature of the micro-dialysis catheter makes the technique described in this study impractical for clinical use and needs to be validated in a more amendable matrix like serum, urine, or plasma. Tandle and colleagues characterized urinary metabolomes associated with GBM patients and applied radiotherapy. They were able to devise a predictive cluster of metabolites with accuracy of 73% in identifying pre-radiation versus post-radiation cohorts. They observed elevation of *N*-acetylated metabolites and TCA cycle intermediates in the post-radiation cohort (66). The findings of this study are consistent with other studies that have identified similar metabolites post-irradiation. For example, elevated *N*-acetylated compounds were observed in the study of Johnson and colleagues of irradiated rat’s urine (61) whereas Wibom’s work reported increase in TCA cycle intermediates (65).

## Future of Radiation Metabolomics with Other Systems Biology Platforms

**Figure 3:**
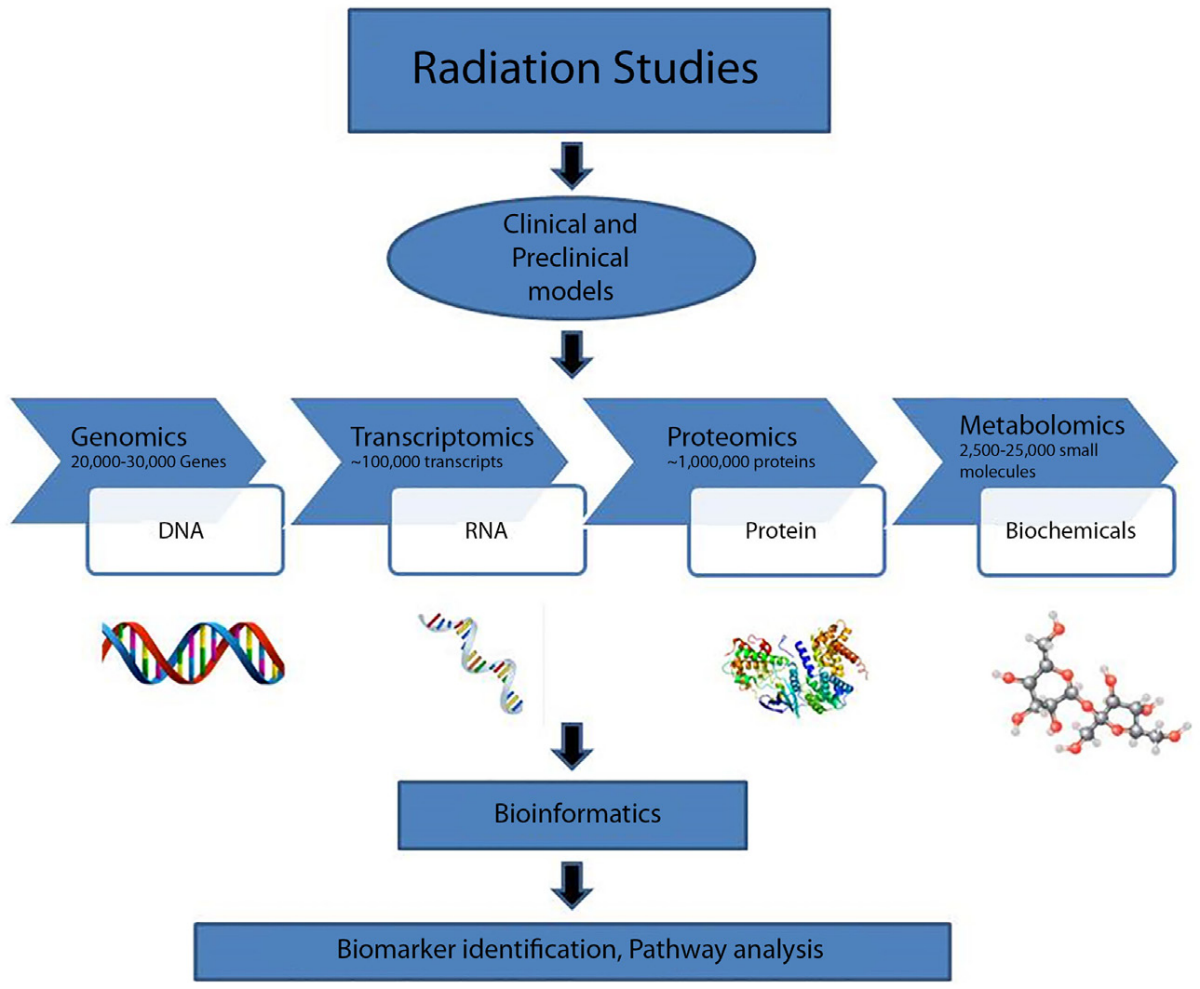
**Systems biology approach to radiation research**.

“Systems biology” has developed in popularity, although the term has been used broadly and its definition is dependent on context (67–70). Large scale studies have focused on radiation therapy acute and late effects. Such investigations contribute to the systems approach and help to establish the clinical efficacy of metabolomics in radiotherapy dosimetry (Figure 3). Systems biology-based studies may facilitate prospective design of adaptive clinical trials, where aspects of the trial can be modified based on analysis of data (71). Furthermore, bio-fluid samples may be collected and stored until patients with similar characteristics and therapy exposures are available for comparison.

Systems biology can be defined as one of the two types (72). Type I systems biology is a term used to describe collecting large amounts of data and analyzing the data together (73). In practice, principles of Type I systems biology is already being applied to radiation biology: although beyond the scope of this review, studies of the consequences of radiation on the genome or transcriptome (74, 75) and proteome (76, 77) are reported in the literature. Although technology seems to have advanced enough to generate broad Type I systems biology-level profiling of patients at many molecular and phenotypic levels, such in-depth profiling in the clinic has yet to be greatly applied in radiation research, or in any other field. A Type II systems biology approach models networks as complex systems by applying principles of systems theory (72). Both Type I and Type II systems biology approaches require extensive computational, statistical, mathematical, and bioinformatics techniques and further advancements in the field of bioinformatics and computational biology. In future, profiling and generation of data at multiple molecular and phenotypic levels may become clinically available in order to facilitate a “systems-level” health status evaluation for the general population (78).

Use of advanced mathematical, statistical, and computational modeling for evaluation of large datasets generated from multiple clinical and molecular parameters to understand radiation biology has already been advanced (79, 80) and, at least at an elementary level, implemented in practice (70). For example, the clinical cooperation group “CCG Personalized Radiotherapy in Head and Neck Cancer” includes the Research Unit of Radiation Cytogenetics at Helmhotz-Zentrum Munchen and the Radiation Oncology Clinics of the Ludwig-Maximilians Universitat Munchen, reports applying systems biology practices to collect genomic copy number profiles, HPV status typing, miRNA profiling, and genetic analysis from cohorts to identify candidate radiation sensitivity modulators for improved radiation therapy of head and neck squamous cell carcinoma. Integrated analysis of these heterogeneous datasets is then applied to models of cell culture to perform “perturbation experiments, network reconstruction, and modeling of radiation-response” (70). In this state-of-the-art translational approach for improving radiation therapy, metabolomics is not one of the platforms used. As promising as metabolomics has been for making basic science discoveries, the field is fertile for translating and integrating data from many systems biology platforms with metabolomics studies to advance radiation dosimetry, tailor radiotherapy, and generate basic knowledge for the improvement and protection of human health.

## Conclusion

Technology continues to drive the metabolomics field; given the plethora of reported studies that use LC-MS, it is reasonable to assume that this is the technological platform of choice for metabolomic data acquisition based on ease of use and accuracy. Moreover, multivariate analysis of large datasets is still evolving, using PCA and support vector machines to identify markers of interest. Metabolomics studies have, in part, validated classical pathways of radiation damage, including oxidative stress and subsequent DNA breakdown. Additionally, PUFA metabolism is often disrupted as an inflammatory effect of radiation exposure. Radiation metabolomics especially with clinical cohort studies is still in nascent stages. Clearly there is a great need for development of biomarkers that would have immediate clinical relevance; these biomarkers could be used for patient triage in a radiological situation as well as for predicting response or non-response to radiation therapy.

In summary, technological advances in detection, acquisition, and processing have made the metabolomics platform a reliable source of data collection, there is still however, an urgent need for standardization of protocols and analytical methods to validate these biomarkers and enable their use in the clinical or translational science settings. Careful and systematic collection, processing and storage of bio fluids are critical for downstream metabolomic studies for validation of research findings across institutions and for future systems biology analyses.

## Author Contributions

AC, AD, and SG conceived the paper while SM, MU, SR, MC, NA, and RU contributed in formulating the figures and the different sections of the review paper.

## Conflict of Interest Statement

The authors declare that the research was conducted in the absence of any commercial or financial relationships that could be construed as a potential conflict of interest.
